# Epidemiology, molecular typing, microbiome-immune interactions and treatment strategies of endometrial cancer: a review

**DOI:** 10.3389/fimmu.2025.1595638

**Published:** 2025-06-25

**Authors:** Bingyan Liang, Jia Tan, Jia Li, Xiaolan Wang, Genlin Li, Huanhuan Li, Ting Li, Hong Gao

**Affiliations:** ^1^ School of Nursing, University of South China, Hengyang, Hunan, China; ^2^ Scientific Research Department, The Second Affiliated Hospital, Hengyang Medical School, University of South China, Hengyang, Hunan, China; ^3^ Department of Neonatology, The First Affiliated Hospital, Hengyang Medical School, University of South China, Hengyang, Hunan, China; ^4^ Operating Room, The First Affiliated Hospital, Hengyang Medical School, University of South China, Hengyang, Hunan, China; ^5^ Center for a Combination of Obstetrics and Gynecology and Reproductive Medicine, The First Affiliated Hospital, Hengyang Medical School, University of South China, Hengyang, Hunan, China; ^6^ Department of Obstetrics and Gynecology, The Second Affiliated Hospital, Hengyang Medical School, University of South China, Hengyang, Hunan, China; ^7^ Ottawa Hospital Research Institute, The Ottawa Hospital, Ottawa, ON, Canada

**Keywords:** endometrial cancer, reproductive tract microbiome, immune response, inflammation, dysbiosis

## Abstract

This review focuses on the field of endometrial cancer. Since 2020, there have been 417,367 new cases of endometrial cancer diagnosed globally and 97,370 deaths reported. Endometrial cancer ranks second in terms of incidence among female genital malignancies and third in terms of mortality among gynecological cancers. The stage, grade, and histological subtype of endometrial cancer were closely correlated with the risk of recurrence and prognosis for survival. Meanwhile, endometrial cancer exhibits significant biological heterogeneity. The complex interactions among the reproductive tract, host cells, and the microbial environment may harbor novel disease mechanisms. In this review, we provide an overview of the epidemiological characteristics, major risk factors, histological and molecular subtypes of endometrial cancer, as well as explore the associations between the female reproductive tract microbiome, immunity, and cancer progression. We also identify the specific roles of different cytokines in the pathophysiology of endometrial cancer. By integrating findings from diverse research fields, this comprehensive review offers an in-depth understanding of the multidimensional nature of endometrial cancer and highlights the significant potential and promising avenues that microbiological factors present for advancing future cancer research and guiding the development of innovative therapeutic strategies.

## 1 Introduction

Endometrial cancer (EC), a common gynecological malignancy, originates from the epithelial lining of the endometrium. Currently, a total of 417,367 newly diagnosed cases have been reported globally, with staggering death toll of 97,370 in 2020 reached, underscoring its significant impact on public health (1). It ranks as the fourth most prevalent malignancy among women in Europe and North America, trailing only breast, colorectal, and lung cancers, with the highest incidence observed in women aged 65–75 years (2). The elevated incidence of EC in Europe and North America may be correlated with the heightened prevalence of obesity and metabolic syndrome in these regions (3, 4). Furthermore, demographic shifts including population aging (5, 6) and the ongoing decrease in fertility rates have partially attributed to the rise in EC prevalence. Notably, obesity is recognized as an independent risk factor for EC, emphasizing its pivotal role in the etiology of this malignancy.

In China, the incidence of EC ranks second among malignancies of the female reproductive system, while its mortality rate stands as the third highest among gynecological malignancies (7, 8). This highlights the substantial impact of EC on women’s health in China. Since the mid-1970s, survival rates for most common cancers have improved, except for cervical cancer and EC, indirectly indicating a lack of significant therapeutic progress in treating these two types of malignancies. Previously, variations in histomorphological classification of EC, combined with limited reproducibility, imprecise risk stratification, and diverse treatment approaches, have contributed to the deficiencies of current risk stratification systems, thereby resulting in lower accuracy in identifying patients at high risk for disease recurrence or metastasis. However, grounded on the seminal discovery of four molecular subtypes of EC by the Cancer Genome Atlas (TCGA), an optimized assessment of disease staging and prognosis has been facilitated at the molecular level, enabling a more precise and individualized approach to EC patient management. Although the TCGA study findings present a promising new prospect for the clinical management of EC, their widespread implementation in clinical practice remains challenging due to the inherent complexity of the molecular subtyping system. This underscores the necessity for further validation and simplification of these findings to ensure their accessibility and practicality in routine clinical settings.

The intricate pathogenesis of cancer has long presented a major challenge facing the entire human race. So far, there have been many hypotheses about the EC pathogenesis. Among them, the estrogenic hypothesis, which posits the pivotal role of estrogen in EC development, has gradually emerged as the prevailing theory. Nevertheless, advancements in gene sequencing technologies have led to the gradual recognition of the uterus as an organ characterized by low microbial abundance. Given the high biological heterogeneity observed in EC, its diverse stages, grades, and pathological tissue types may exhibit associations with recurrence rates and prognostic survival, potentially revealing an alternative underlying mechanism involving interactions among the genital tract, host cells, and the microbial environment. This insight has inspired our comprehension of the relationship between the EC occurrence and the genital tract microbiome. At present, research on the relationship between the microbiome and the immune system remains in its early stages, and there are significant deficiencies in comprehensive studies linking molecular subtypes with immune microenvironment analysis. Regarding the mechanism by which microbial metabolites regulate cytokines in the body and affect tumor immunosurveillance, there is still controversy (9, 10). Therefore, a dedicated endeavor is warranted to elucidate the causal relationship between the composition and distribution of the genital tract microbiome and the host’s health status, with the aim of further enhancing our comprehension of the intricate interplay between these factors. From a microbial perspective, this review systematically integrates the application of epidemiological patterns, molecular subtype frameworks, and the interactions between the microbiome and immune microenvironment in EC, providing a comprehensive understanding of their roles in disease progression. It primarily elucidates the diagnostic potential of microbial biomarkers, explores the underlying pathogenic mechanisms, and evaluates the feasibility of therapeutic interventions targeting the microbiome.

## 2 Epidemiology

The epidemiological characteristics of EC exhibit pronounced variations across racial, geographical, and socioeconomic dimensions, with a particularly noteworthy high prevalence in developed countries (11). There was obvious regional heterogeneity in the age-standardized incidence rate (ASIR) of EC (**Table 1**). The incidence rates of EC varied significantly among different racial groups, exhibiting distinct trends. From 1900 to 2017 in America, the average incidence of EC was estimated at 25.9 per 100,000 among white women, 25.0 per 100,000 among all women, and 21.0 per 100,000 among black women (12). During the period from 2000 to 2015 in the United States, the incidence of EC was the highest among non-Hispanic whites, but the annual percentage change (APC) remained relatively stable at approximately 0.3%. And the incidence rates of EC among non-Hispanic blacks (APC = 2.0%), Hispanics (APC = 2.6%), and Asian/Pacific Islanders (APC = 1.9%) changed significantly and showed a continuous upward trend (13). This indicated the huge differences and inequalities among races and might imply the significant challenges faced by low socioeconomic groups. Based on the aggregated analysis of epidemiological research data in the United States from 2010 to 2019, it was found that the annual increase in EC mortality among African American/Black individuals was 1.9%, whereas for American white women, the average annual increase was 1.6% (14). Further studies have shown that the higher EC mortality in black women compared to white women can be attributed to tumor genomic differences, less access to chemotherapy and radiotherapy, low socioeconomic status, and lack of health insurance (15). Black women are more likely to present with advanced stages and higher grades of EC, as well as a more aggressive histomorphological subtype of the disease (15–17). The disparities in survival outcomes caused by race differences highlight the critical need for early screening and targeted interventions tailored to specific groups. It was worth noting that the median age at diagnosis for EC patients was 63 years old, and women over 50 years old accounted for a predominant majority of approximately 90% of all cases. The age distribution of disease onset showed a distinct pattern. Specifically, only 14% of EC cases were diagnosed prior to menopause, and an even smaller proportion, 5% involved women under the age of 40 (15, 18, 19).

**Table 1:** Comparison of endometrial cancer incidence in different countries/regions.

Countries/Regions	Time point 1	Age standardized incidence rate^*^	Time point 2	Age standardized incidence rate^*^	Growth rate(%)^**^
China	2004	6.20	2019	10.28	65.81
Western Europe	1990	40.0	2019	82.5	106.25
Australasia	2000	10.9	2019	11.3	3.67
Switzerland	1960	16.2	2016	28.6	76.54
Korea	1999	2.38	2018	7.29	206.30
Southeast Asia	1990	7.1	2019	22.1	211.27
Netherlands	2004	17.5	2018	17.2	-1.79
the United States	2000	11.4	2019	13.8	21.05

*The data is expressed as the age-standardized incidence rate of endometrial cancer per 100,000 people per year.

**The growth rate is calculated and determined from Time point 1 to Time point 2. Time point 1 is the initial value and time point 2 is the final value.

$Formula:Growth\;Rate=(\frac{\text{Final Value-Initial Value}}{\text{Initial Value}})\times 100$ .

Morbidity, recurrence rate, and mortality of EC exhibited a negative correlation with socio-economic indicators; that is, regions with lower socioeconomic levels tend to have higher EC morbidity and mortality (20). This relationship significantly increases the disease burden in low- and middle-income countries, posing a substantial threat to the health and well-being of the population (21). Significantly, limited accessibility to healthcare resources and a prevalent lack of awareness regarding health maintenance have contributed to an increase in late-stage cancer diagnoses upon initial detection, accompanied by a markedly shorter survival duration for individuals with lower socioeconomic status. This phenomenon underscored the urgent need for heightened attention from all sectors of society and the adoption of effective intervention measures (22, 23).

EC shows significant variations across dimensions such as race, geography and socio-economic factors. The epidemiological differences may be associated with EC and microbiome, potentially involving underlying biological mechanisms.

## 3 Risk factor

### 3.1 Metabolic syndrome

#### 3.1.1 Obesity

According to the American Cancer Society, approximately 70% of EC can be attributed to overweight and physical inactivity, with obesity accounting for 57% (24). Obesity is a significant risk factor, and its association with EC is particularly prominent among all gynecological cancers. A robust positive correlation between obesity and EC has been consistently demonstrated (25, 26). As the body mass index (BMI) increased, the relative risk (RR) of developing EC also significantly rised (27). Setiawan et al. reported 10 cohort studies and 14 case-control studies in the meta-analysis to explore the risk factors of endometrial cancer. Among them, 77.7% of EC patients were white women with an average age of 62 years, and the proportion of postmenopausal women was as high as 79.6%. The study population came from the United States, Canada, Europe and Australia. When analyzing the association between BMI and EC, it was found that the RR for overweight individuals (BMI between 25kg/m² and 29.9kg/m²) was approximately 1.5; for first-degree obesity(BMI between 30kg/m² and 34.9kg/m²), the RR increased to 2.5; for second-degree obesity (BMI between 35kg/m² and 39.9kg/m²), the RR significantly increased to 4.5; for third-degree obesity (BMI ≥ 40kg/m²), and the RR markedly rose to 7.1 (28). These findings indicated the influence of BMI on type I tumors was greater than that on type II tumors. In a prospective cohort study conducted in Japan, 36,172 participants aged 40–59 years were enrolled. The results demonstrated that there was no significant association between BMI and the risk of type II EC (hazard ratio[HR] = 133%, 95%CI: 0.74-2.38) (29). Recent data suggested that obesity was driving the age of obesity-related cancers to younger age groups, particularly in EC, where there was a sustained and significant upward trend in age-specific incidence among women aged 50 years and younger (30, 31). Obesity constitutes a chronic inflammatory condition characterized by the establishment of a pro-inflammatory environment, primarily mediated through increased secretion levels of circulating biomarkers such as c-reactive protein, interleukin-6, and tumor necrosis factor-alpha (32). The main mechanism by which obesity promotes EC development was the aromatization of adrenal androgens in the surrounding adipose tissue into estrogen, resulting in a substantial elevation of estrogen levels in the body. These high levels of estrogen persistently stimulated the endometrial tissue, potentially leading to abnormal hyperplasia and even cancer of the endometrial tissue (33–35). For postmenopausal women, they faced dual risks: on the one hand, due to the lack of natural progesterone in the body, obesity exacerbated the excess state of non-antagonistic estrogen; On the other hand, their immune function tends to decline with advancing age (34). The combination of these two factors greatly increased the risk of EC.

#### 3.1.2 Diabetes mellitus

Numerous studies have consistently established a positive correlation between diabetes and the risk of EC(**Table 2**). The association between diabetes and increased EC risk may involve multiple intricate mechanisms, including obesity, hyperglycemia, hyperinsulinemia, activation of the Insulin-like Growth Factor-1 (IGF-1) pathway, and elevated levels of inflammatory cytokines (36–38). High levels of blood glucose can induce angiogenesis and lead to significant changes in the concentration and types of immune cells as well as inflammatory factors (39). A meta-analysis conducted in 2007 demonstrated that diabetic patients had a twofold increased risk of developing EC compared to individuals without diabetes (40). Despite long-standing controversies regarding the comorbid impact and confounding factors between diabetes, obesity, and EC, comprehensive analyses of four BMI-adjusted cohort studies have confirmed diabetes as an independent risk factor for EC (41–43). Among them, patients with EC who have type 2 diabetes exhibited an augmented risk of mortality (44).

**Table 2:** Relationship between diabetes and the risk of endometrial cancer.

Study (first author, et al., year)	Territory	Design	Sample size (cases/non-cases)	Sample age	Time apan	Risk assessment index(RR/OR/SIR, 95% CI)	Main conclusion
Massouh, et al., 2024 (169)	America	Cross-sectional	106/302	≥18	2020	OR=1.54(1.01-2.34)	Women with diabetes have twice the risk of developing EC compared to those without diabetes.
Esposito, et al., 2021 (170)	Italy	Case-control	454/908	18-79	1992-2006	OR=0.45(0.28-0.73)	A diet that reduces the risk of diabetes can also lower the risk of developing EC.
Zahuliene, et al., 2021 (171)	Lithuania	Cohort	995-76113	≥40	2000-2012	SIR=1.69(1.59-1.80)	Compared with ordinary women, the risk of EC for diabetic women is significantly increased.
Kim, et al., 2020 (172)	Utah	Cohort	2314/8583	≥18	1997-2012	HR=2.99(2.59-3.45)	Survivors of EC are more prone to developing diabetes.
Wartko, et al., 2017 (173)	Washington	Case-control	593/5743	≥15	1987-2013	OR=1.73(1.27-2.35)	Early-onset EC is associated with gestational diabetes in young women.
Luo, et al., 2014 (174)	America	Cohort	1241/86866	50-79	1998-2010	HR=1.44(1.13-1.85)	Diabetes is positively correlated with the incidence of EC, but it is affected by body weight.

(1) Sample size is presented as cases/non-cases for case-control studies, events/non-events for cohort studies, and affected/unaffected for cross-sectional studies.

RR, Relative Risk; OR, Odds Ratio; SIR, Standardized Incidence Rate; CI, Confidence Interval; EC, Endometrial Cancer.

#### 3.1.3 Polycystic ovary syndrome

**Figure 1:**
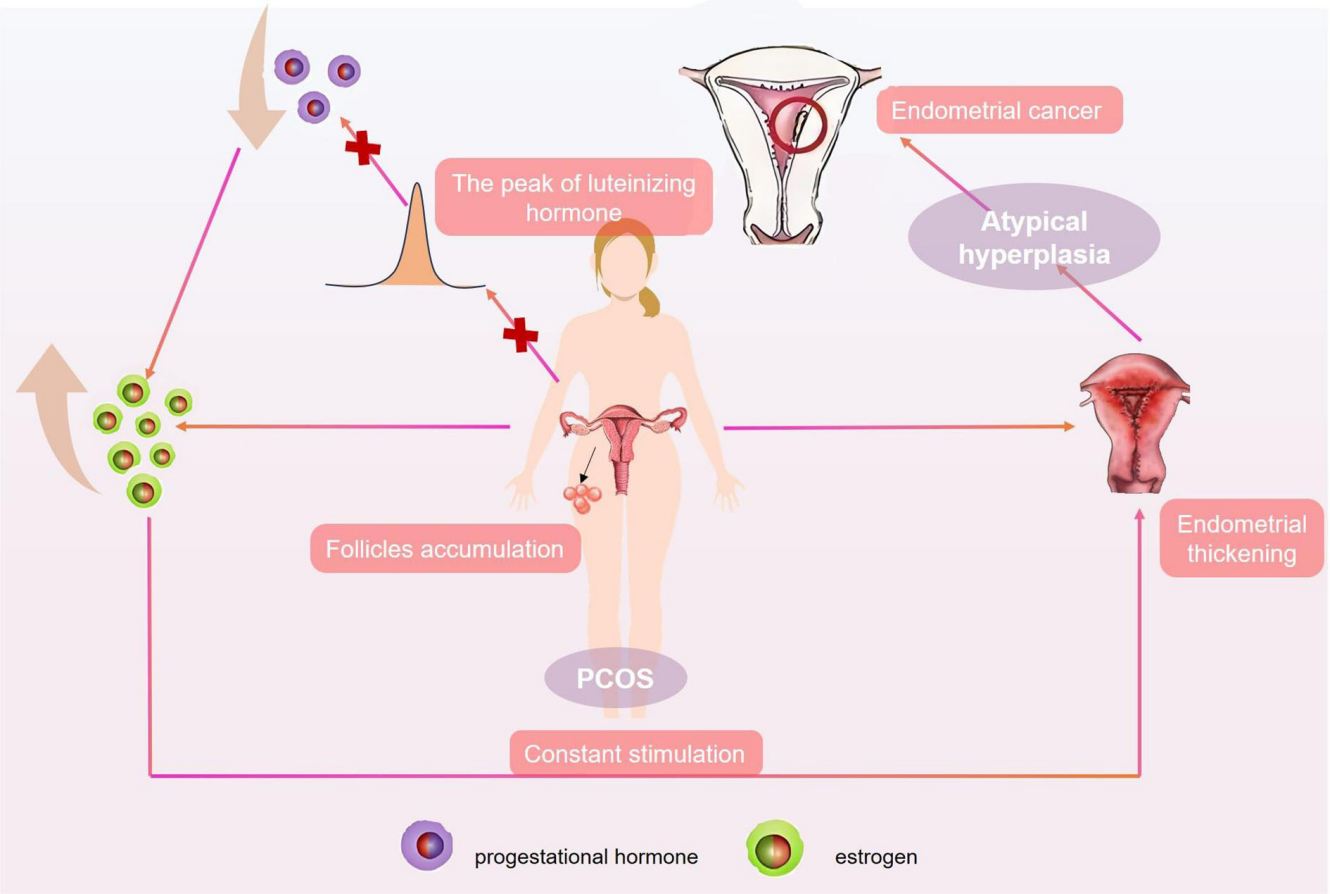
Polycystic ovary syndrome is associated with an increased risk of endometrial cancer in women. Patients with polycystic ovary syndrome often have abnormal levels of estrogen. Their follicles are difficult to mature and ovulate, resulting in the accumulation of small follicles in the ovaries and excessive secretion of estrogen. The absence of a luteinizing hormone peak hinders the formation of corpus luteum, leading to an imbalance in estrogen and progesterone secretion. The endometrium undergoes prolonged stimulation by elevated levels of estrogen, leading to sustained proliferation. Without the antagonism of progesterone, the endometrium fails to undergo the normal periodic exfoliation and instead continues to thicken, which may develop into atypical hyperplasia and increase the risk of EC.

Polycystic ovary syndrome (PCOS) is a heterogeneous endocrine disorder. Substantial research has demonstrated an elevated risk of EC among women with PCOS (45–47). This heightened risk stemmed from the typical association of PCOS with anovulation, which manifested as a persistent dominance of estrogen levels. This sustained estrogenic stimulation led to prolonged endometrial proliferation, thereby increasing the risk of EC(**Figure 1**). An early meta-analysis, which encompassed four case-control studies involving a total of 4,056 women, revealed that individuals with PCOS had a nearly three-fold increased risk of developing EC compared to the general population (odds ratio [OR]: 2.70; 95% confidence interval [CI]: 1.00-7.29) (48). The correlation was subsequently demonstrated in a later meta-analysis (49).

It has been confirmed that PCOS is closely related to systemic and local low-grade inflammatory reactions (50). Existing data showed significant changes in the composition of immune cells among PCOS patients, including an increase in the number of macrophages and a decrease in the density of uterine natural killer cells (uNK) in the endometrium, which may affect immune homeostasis and endometrial receptivity (51, 52).

### 3.2 Other Factors

Tamoxifen is a critical drug widely used in endocrine therapy and for the prevention of hormone receptor-positive breast cancer. Its unique mechanism of action involved exerting anti-estrogenic effects on breast tissue while producing pro-estrogenic effects on the uterus (53). Long-term use of tamoxifen was associated with an increased EC risk, particularly in postmenopausal women. This risk was further exacerbated in patients who already have endometrial lesions, such as abnormal uterine bleeding or endometrial thickening (54–56). It is noteworthy that the majority of tamoxifen-induced EC cases were detectable at an early stage, and thus the overall prognosis was favorable (57). However, with prolonged duration of tamoxifen treatment, the risk of EC also escalates, potentially leading to more severe prognostic outcomes for patients. A study specifically indicated that patients treated with tamoxifen for 2 to 5 years had a relative risk of 2.2 (95% CI: 1.2-3.2) for EC compared to those not receiving tamoxifen treatment, while those treated for at least 5 years faced an even higher risk of 6.9 (95% CI: 2.4-19.4) (58). Another case-control study reported similar findings (59). Compared with patients not receiving tamoxifen therapy, patients treated with tamoxifen exhibited a significantly elevated risk of EC (OR = 2.4; 95%CI = 1.8-3.0). Moreover, the risk of EC increased progressively with longer durations of tamoxifen treatment(*P* < 0.001). Specifically, patients who received tamoxifen treatment for more than 5 years had an OR of 3.6 (95%CI = 2.6-4.8) (59). Both this study and prior research have consistently demonstrated a significant association between prolonged tamoxifen use (exceeding 5 years) and an increased risk of EC. Close attention should be paid to the condition of endometrial cancer in female patients with negative estrogen receptors during tamoxifen treatment (both premenopausal and postmenopausal) and for at least 5 years after the treatment. In addition, Lynch syndrome is a genetic disorder with an autosomal dominant inheritance mode (60). It is not only closely related to the occurrence of hereditary colorectal cancer (61), but also significantly associated with an increased risk of EC (62, 63). Lynch syndrome is primarily characterized by germline mutations in the DNA mismatch repair (MMR) system genes, including MSH2, MLH1, MSH6, and PMS2 (64). These genes play a crucial role in recognizing and repairing errors occurring during DNA replication. Mutations in MMR system genes resulted in dysregulation of intracellular immunity or abnormal cytokinesis. It interfered with the normal growth and division processes of cells, thereby significantly raising the risk of EC development. Of note, hereditary EC tends to exhibit an earlier onset compared to sporadic EC. That is to say, individuals with Lynch syndrome are at a higher risk of developing EC at a younger age (65–67).

## 4 Comparative analysis of tissue and molecular typing in endometrial cancer

### 4.1 Histological grading and typing

In 1973, the histological classification of EC was established by the Fédération Internationale de Gynécologie et d’Obstétrique (FIGO), classifying it into three grades: well-differentiated EC(G1), moderately differentiated EC (G2), and poorly differentiated EC (G3) (68). In 1983, Bokhman proposed a traditional binary model that classified EC into type I and type II (69). Type I EC, known as endometrioid adenocarcinoma, includes G1 and G2 subtypes. This type of cancer is estrogen-dependent and has been associated with obesity and excess estrogen (70, 71). Type II EC, referred to as non-endometrioid adenocarcinoma or non-estrogen-dependent carcinoma, encompasses G3 endometrioid carcinoma and non-endometrioid carcinoma, such as representative uterine papillary serous carcinoma (UPSC), clear cell carcinoma (CCC), and undifferentiated carcinoma (UDC) (72). In 2014, the EC classification by the World Health Organization (WHO) primarily focused on histomorphological features, categorizing it into distinct subtypes including endometrioid carcinoma, mucinous carcinoma, serous carcinoma, clear cell carcinoma, carcinosarcoma, neuroendocrine carcinoma, mixed cell adenocarcinoma, and undifferentiated carcinoma (73). In 2020, an updated version of the classification system for EC was introduced, formally incorporating renal adenocarcinoma, renal adenoid cancer, squamous cell carcinoma (im), and mucus carcinoma. Additionally, neuroendocrine tumor was separated from the original EC chapter and established as a distinct chapter focusing on neuroendocrine tumors of the female genital tract (74).

The WHO classification system is widely regarded as the “gold standard” in the field of EC. However, due to the limited global accessibility of certain molecular techniques, traditional histocytology continues to serve as the fundamental approach for classifying EC and remains the primary guideline for the clinical management of patients (75). In conditions permitting, the WHO’s new classification system emphasizes that immunohistochemical technique is highly recommended to complement histologic diagnosis for distinguishing between endometrial carcinoma and other types of endometrial malignancies, as this approach holds particular significance (76). As we continue to explore the histological aspects of EC, it is expected that future editions of the WHO classification will undergo further advancements.

### 4.2 Molecular typing

In 2013, the Cancer Genome Atlas (TCGA) research marked a profound shift in cancer classification from the traditional morphological basis to the molecular level. Based on distinct mutational profiles, EC have been classified into four major categories: POLE (DNA polymerase) mutant, microsatellite instability (MSI) high mutant, low copy number (CN-L), and high copy number (CN-H). Among these four types, POLE mutants exhibit the most favorable prognosis, while the CN-H subtype portends the worst (77).

In retrospect, despite the widespread adoption of the traditional binary staging system proposed by Bokhman in 1983 and the histopathological staging introduced by WHO in 2014, both have failed to adequately capture the inherent molecular heterogeneity in malignancies, thereby limiting their utility as accurate references for predicting patient clinical outcomes. Especially in type I EC, the phosphatase and tensin homolog (PTEN) gene mutations are among the most prevalent molecular events (77). PTEN, which acts as a tumor suppressor gene, encodes a protein that crucially regulates cell growth through modulating tyrosine kinase activity. Mutations in this gene lead to uncontrolled cellular proliferation (78). Additionally, Kras proto-oncogene aberrations played a pivotal role in the initiation and progression of type I EC by disrupting the regulatory mechanisms of normal cell division, further fueling tumorigenesis and development (78).

The TCGA classification is a clinically-oriented method that shows better prognostic relevance and optimized inter-observer consistency when compared to traditional morphological classification. The traditional classification of EC provides a specific research framework. However, it exhibits obvious limitations in terms of subtype classification uncertainty, prognostic variability, homogeneity, and the correlation with other factors contributing to endometrial cancer. Molecular classification represents a groundbreaking advancement in the realm of EC classification research, providing an important theoretical basis for personalized treatment strategies. With the deepening comprehension of the four different molecular subtypes of EC identified by TCGA, the related molecular genetic changes and clinicopathological significance have become increasingly important. To streamline operational procedures and enhance clinical feasibility, subsequent research has simplified the TCGA molecular classification by dividing EC into four distinct groups: POLE mutation (POLE mut), Mismatch Repair Deficient (MMRd), Non-Specific Molecular Profile (NSMP), and p53 Abnormality (p53abn). Within this simplified framework, POLE mut signified a favorable prognosis, while MMRd and NSMP indicated intermediate prognoses (79). Conversely, p53abn denoted an unfavorable prognosis (79). The proposed classification method demonstrated the potential to accurately predict the prognosis of EC and enhance the treatment effect.

To sum up, EC is a group of highly heterogeneous tumors, and different types of EC have distinct histological features, molecular patterns, and clinical significance. Therefore, there is an urgent need to develop novel molecular staging strategies based on traditional staging in order to achieve more precise diagnosis and treatment.

## 5 The human microbiome and cancer

The most prominent hypotheses for EC are obesity and excessive estrogen, while the potential role of reproductive tract microbiome in EC has not received adequate attention (80). As a result, the microbiome is often referred to as the forgotten organ. Indeed, both hormones and inflammation play an extremely important role in the pathogenesis of EC, constituting a complex equilibrium relationship. It is hypothesized that dysbiosis of the microbiota could alter immune and metabolic signaling, thereby exerting influence on various cancer-related features including chronic inflammation, epithelial barrier disruption, changes in cell proliferation and apoptosis, genomic instability, angiogenesis, and metabolic dysregulation (81). Currently, the most widely accepted link between microbial communities and cancer lies in their intricate influence on the host’s immune system. Specifically, microorganisms play a vital role in activating, shaping, and regulating the host’s immune responses. These microbes are capable of stimulating immune reactions. However, in cases of microbial community imbalance (termed dysbiosis), they may trigger the abnormal secretion of a series of pro-inflammatory cytokines or growth factors. These factors, under certain conditions, were believed to contribute to the development of cancer (82).

Existing studies have proven that chronic inflammation caused by the microbiome plays an important role in the occurrence, development, and metastasis of cancer (83–86). For instance, chronic gastritis caused by Helicobacter pylori was closely related to gastric adenocarcinoma (87); human papillomavirus infection made local tissues more susceptible to the invasion of pathogenic microorganisms, thereby inducing inflammatory reactions, which was closely related to cervical cancer (88); chronic inflammation and fibrosis of the liver caused by hepatitis viruses (types B and C) are closely related to liver cancer (89) (**Table 3**). At the same time, the carcinogenic mechanisms of these infection-associated cancers have been extensively studied, particularly focusing on the impact of viral and bacterial infections as well as resulting inflammation on cell proliferation, cell signaling, and genetic alterations. A rich microbiota has been detected in the reproductive tract of patients with endometrial hyperplasia and cancer, suggesting that these bacteria may play an infectious/inflammatory role in the onset of EC (90).

**Table 3:** The association between diverse microbes and cancer.

Microbes	Cancer type	The role of microbes	Underlying mechanisms	References
*Helicobacter Pylori*	Gastric carcinoma	Carcinogenic	(1)The pathogenic pedigree variation containing 9 genes is associated with the risk of gastric cancer;(2) *H.pylori* infection significantly interacted with pathogenic variations in homologous recombination genes, elevating gastric cancer risk by over 16-fold (95%CI: 2.22-29.81, *P*=0.02);(3) Its virulence factors triggered chronic gastric mucosal inflammation, progressing to atrophy, intestinal metaplasia, dysplasia, and potentially gastric cancer;(4)It might modulate host immunity and gene expression.	(89, 175)
Human papilloma virus(HPV)	Cervical cancer	Oncogenic	(1)HPV overexpressed E6 and E7 oncoproteins, disrupting the normal functions of host tumor suppressor genes (2); Activate the MALAT1-ALKBH5 signaling axis, regulate the expression of key genes such as MMP2 and MMP9, and promote cell proliferation and metastasis.	(90, 176)
Hepatitis B virus (HBV) and Hepatitis C virus (HCV)	Liver cancer	Proinflammatory; Carcinogenic	(1)HBV altered the boundaries of many topologically associating domains (TADs), potentially facilitating cancer metastasis (2);Lead to the dysregulation of cellular signaling pathways, directly or indirectly causing oxidative stress damage within cells (3);They enhanced the overall proximal chromatin interactions (CIs) in liver cells.	(175, 177)
Epstein-Barr virus (EBV)	Nasopharynx cancer(NPC)	Oncogenic	(1)An antibody named P85-Ab within EBV exhibited high sensitivity (95%CI:86.4-97.8) and specificity (95%CI:97.8-99.9) in the screening of NPC (2);Activate the p62-Keap1-NRF2 signaling pathway and reduce the sensitivity of nasopharyngeal cancer cells to ferroptosis (3);The latent membrane protein 1 (LMP1) encoded by EBV enhances the anti-apoptotic ability of nasopharyngeal carcinoma cells (4);LMP1 induces immune evasion.	(178–180)
Human T cell lymphocytophil virus-1 (HTLV-1)	Leukemia	Carcinogenic	(1)The antisense gene HBZ encoded by HTLV-1 promotes the proliferation of leukemia cells.	(181)
Kaposi sarcoma-associated herpes virus (KSHV)	Kaposi’s Sarcoma (KS)	Carcinogenic	(1)Evading the innate and specific immune responses of the host, thus remaining in a latent state within the host and recurring repeatedly (2);The KSHV protein vFLIP stimulated the activation of NF-kB, which is associated with KS (3);KSHV-encoded proteins could upregulate the expression of VEGF (vascular endothelial growth factor), stimulating angiogenesis.	(182–186)
Schistosoma haematobium	Carcinoma of urinary bladder	Proinflammatory; Carcinogenic	(1)The retention of Schistosoma haematobium eggs in bladder tissues might lead to persistent inflammation, markedly increasing the activity of T-cell populations, especially CD3+, CD4+, and regulatory T cells (Tregs) (2);It elevated the proportion of CD19+, CD24+, CD38+ Bregs and proinflammatory cytokines (IL-1β, IL-6, and TNF-α).	(187)

## 6 Female reproductive tract microbiome and endometrial cancer

### 6.1 Origin of the reproductive tract microbiome

During the early 20th century, there was a prevailing belief that the cervicovaginal epithelium exclusively harbored microorganisms, and it was widely held that a healthy uterus should be a sterile cavity (91). However, in the 21st century, particularly since 2007, the advent of next-generation sequencing (NGS) technology has revolutionized the field. It makes it possible to conduct a comprehensive quantitative assessment of the uterine microbiome, which was previously unattainable using traditional culture-based methods (92). The combination of this technological breakthrough and the availability of bacterial genome sequences has facilitated a deeper understanding of uterine microbial composition, surpassing the limitations of culture-based methods (81). Currently, it is widely acknowledged that the female reproductive tract (FRT) harbors an active microbiome. However, a comprehensive understanding of the potential roles that these microbiomes play in fertility and gynecological diseases remains elusive and merits thorough exploration (93). The composition of the lower genital tract microbiome undergoes dynamic changes throughout a woman’s lifespan, influenced by factors such as age, hormonal variations, sexual activities, and hygienic habits. Considering the FRT as an open system, the microbial communities in the upper reproductive tract exhibit diverse colonization pathways, including ascending microbiota from the lower reproductive tract, bacterial transmission through blood circulation, cervical tube abnormalities, retrograde infection of the uterus by abdominal microorganisms, gynecological surgery interventions, and direct drug inoculation methods (94–97). Among these pathways, one of the most certain and critical colonization pathways is the upward migration of bacteria from the vagina.

### 6.2 Vaginal microbiome and endometrial cancer

In the majority of women in their reproductive years, the microbiome within the lower FRT (including the vagina and cervix) was dominated by various species of *Lactobacillus*. Studies have shown that *Lactobacillus* species, including *L.iners*, *L.crispatus*, *L.gasseri*, and *L.jensenii*, dominate the vaginal microbiome and present five main types of community states (CSTs) (98). In these CSTs, *Lactobacillus* primarily dominated CST I, II, III and V (99). CST IV stands out due to its significant incorporation of anaerobic bacteria, such as *Prevotella*, *Dialister*, *Atopobium*, *Gardnerella*, and *Megasphaera*. This indicated a complex microbiota structure that was skewed towards an anaerobic environment. Given the vast diversity of *Lactobacillus* species and their varying antimicrobial capabilities, this underscored the significant alterations in composition and distribution of the vaginal microbiome that occur under different disease states (98).

Although the current research on the vaginal microbiome in patients with EC is relatively limited, a recent study has yielded significant new findings. Not only did the vaginal microbiome effectively distinguish between benign gynecological diseases and EC, but it also had the potential to predict cancer grade and histological type. Vaginal microbiome samples were prospectively collected from 61 patients with diverse racial and ethnic backgrounds who underwent hysterectomy (100). These patients were categorized into three groups: a control group comprising individuals with benign gynecological diseases, a low-grade EC group, and a high-grade EC group. The results revealed significant differences in both α-diversity and β-diversity among the three groups, and identified four vaginal CSTs associated with disease grade. Specifically, the benign diseases were predominantly clustered in CST1; low-grade EC exhibited a higher concentration in CST2; and high-grade EC was found in both CST3 and CST4 (*P*=0.036) (100). Furthermore, some studies have unveiled significant variations in compositions of cervical-vaginal microbiome among different pathological subtypes of EC. For example, in patients with high-grade EC, the abundances of *Fusobacterium nucleatum* and *Prevotella bivia* were significantly increased. Among them, *Prevotella bivia* has been demonstrated to up-regulate pro-inflammatory factors (such as lysosomal associated membrane protein 3 (LAMP3), STAT1, and TAP1), induce the overactive immune response, and lead to carcinogenesis and poor treatment outcomes (101). *F. nucleatum* can promote tumor growth and metastasis (102, 103). The FadA protein of *F.nucleatum* was capable of binding to the E-cadherin of host cells, which activated signaling pathways, thereby leading to the expression of oncogenic microRNA miR21 and an increase in pro-inflammatory cytokines such as TNF, IL-6, IL-8, and IL-1β (104–106). Once a tumor forms, *F.nucleatum* could bind to tumor cells via its Fap2 lectin, thereby leading to its accumulation within the tumor tissue (107). Conversely, low-grade EC patients exhibited significant increases in the abundances of *Clostridium* spp., *Corynebacterium amycolatum*, *L.gasseri*, and *Peptoniphilus duerdeni*; only *Staphylococcus epidermidis* exhibited a lower abundance (100). The presence of specific bacterial species, such as vaginal *Proteobacteria* and *Porphyromonas*, has been observed to be correlated with a high vaginal pH and the occurrence of EC (9). Another study found that the vaginal and cervical microbial ecology of healthy females was predominantly shaped by several key species, including *L.crispatus*, *L.iners*, *L.gasseri*, and *G.vaginalis (* 108). However, a significant alteration in bacterial frequencies was observed within vaginal samples when focusing on patients with EC (*P*=0.003) (108). The detection frequencies of two specific bacteria, *Mobiluncus curtisii* and *F.nucleatum*, were aberrantly elevated in the vaginal samples of EC patients, with this increase being more pronounced in the vagina compared to the cervix (35.5% vs 17.7%, *P*=0.009; 38.5% vs 24.0%, *P*=0.043) *(* 108). Furthermore, the composition of vaginal microbiome exhibited significant differences among different subtypes of EC. Gressel et al. investigated the characteristics of vaginal microbiome in patients with endometrioid carcinoma and uterine serous carcinoma, revealing a substantial decrease in the alpha diversity of vaginal microbiome in patients with uterine serous carcinoma compared to those with endometrioid carcinoma (Chao1 index, *P*=0.004; Fisher index, *P*=0.007) *(* 109).

These findings confirmed that the composition and abundance of the vaginal microbiome may serve as robust predictors for EC grading, typing, histological features, and diagnosis, providing a novel microbiological perspective.

### 6.3 Endometrial microbiome and endometrial cancer

Indeed, the specimen collection process for the endometrial microbiome often requires invasive or somewhat traumatic procedures, necessitating a heightened awareness of potential contamination during sampling. Additionally, the complexity introduced by factors such as the inherent cyclical changes of the endometrium, patient age, and ethnicity should be taken into consideration. These technical challenges and diverse influencing factors have in turn limited the scope of research on the endometrial microbiome. Nevertheless, it is noteworthy that despite these constraints, existing studies focusing on specific populations (e.g., infertility patients, EC patients) or under particular conditions (such as pre- and post- assisted reproductive technology treatments) have robustly demonstrated the crucial role of endometrial microbiome in the initiation and progression of EC. The microbial community of the endometrium exhibited high microbial diversity, yet its overall relative abundance remained relatively low (9, 80, 110). According to the currently available data, it has been observed that the dominant microbial phyla in the uterine primarily consist of Firmicutes, Bacteroidetes, Proteobacteria, and Actinobacteria (81). Most studies have indicated that the Firmicutes phylum dominates, with Lactobacillus being a significant genus whose importance cannot be overlooked. This recent finding is highly consistent with previous in-depth investigations into the vaginal microbiome (111, 112). Compared to the benign endometrial tissues, the microbial α-diversity in the endometrium of EC patients was significantly elevated (*P*=0.04). Additionally, there was a notable decrease in *Lactobacillus* abundance and a significant increase in specific bacterial taxa such as *Anaerobic cocci*, *Porphyromonas*, *Prevotella*, *Fusobacterium*, *Acidobacteria*, Firmicutes, Spirochetes, Actinobacteria, and Proteobacteria (9, 113). Significantly, *Bacteroides* and *Faecalibacterium* exhibited a strong association with the EC patient cohort. The abundance of *Bacteroides* increased in the mucosa-associated lymphoid tissues of cancer patients and triggered immune responses, which may be closely related to the poor prognosis of cancer lesions (114–116). Besides, microorganisms belonging to the genera *Porphyromonas* and *Atopobium* exhibited a high prevalence in EC samples, whereas they were virtually absent in normal endometrial samples (9). The abundance of *Micrococcus* on the endometrium of EC patients was significantly higher compared to benign uterine lesions (117). The abundance of *Micrococcus* was positively correlated with the levels of IL-6 and IL-17 mRNA in endometrial tissues, thereby inducing excessive expression of immune response (117). Further speculation suggested that these abnormal alterations in the reproductive tract microenvironment, particularly the increased microbial diversity and the enrichment of specific pathogens, might contribute to the onset and progression of endometrial inflammation. The persistent inflammatory state of the endometrium, as a crucial risk factor for EC, could facilitate cancer cell proliferation and invasion through various mechanisms, thereby accelerating cancer progression (118, 119). Moreover, notable differences in endometrial microbiome were observed between EC tissues and their adjacent non-cancerous tissues. A study has conclusively demonstrated that, compared to non-cancerous tissues, the endometrial microbial communities within cancer tissues exhibit a significant increase in both α-diversity and evenness (*P*<0.01), highlighting the intricate relationship between the endometrial microbiome and the initiation and progression of EC (120).

These findings underscored the significance of the endometrial microbiome in the onset, progression, diagnosis, and prognosis of EC. An increase in the diversity of endometrial microbial communities and the enrichment of specific pathogens may both contribute to the initiation and progression of EC.

## 7 Immunity, endometrial microbiome, and endometrial cancer

### 7.1 Immune response of the vaginal microbiome in endometrial cancer

The interaction between bacteria and their hosts involves the engagement of pattern recognition receptors (PRRs), including dectin-1 receptors, toll-like receptors (TLRs), and nucleotide-binding oligomerization domain (NOD)-like receptors. These PRRs are widely distributed on both squamous epithelial cells of the vaginal lining and columnar epithelial cells of the FRT. They play a crucial role in facilitating the recognition and sensing of microorganisms by host cells, thereby influencing the survival of these bacteria within the reproductive tract. Essentially, the survival of commensal bacteria in the FRT is intimately linked to the host’s ability to develop immunological tolerance towards them.

In recent years, numerous *in vitro* and *in vivo* studies demonstrated that dysbiosis of the vaginal microbiome could disrupt local microhomeostasis and alter immune parameters (including immune cells and cytokines), thereby inducing pro-inflammatory responses. A recent *in vitro* study found that certain bacteria in the reproductive tract had the ability to directly induce the expression of pro-inflammatory cytokines, such as vaginae and micromonas inducing pro-inflammatory cytokines IL-1α, IL-1β, IL-17α, and TNF-α. Furthermore, they also altered the transcription of CCL13, CCL8, CXCL2, IL22, and IL9 transcripts (121).

Cytokines represent a class of small soluble proteins synthesized by various cells types in response to infection and inflammation. For instance, interleukin-6 (IL-6), interleukin-8 (IL-8), and interleukin-17 (IL-17) are pro-inflammatory cytokines that play a crucial role in inflammation and tumorigenesis. Notably, IL-6, IL-8, and IL-17 have been proven to be associated with EC. There were significant differences in the mRNA expression of these cytokines between women with EC and those with benign uterine lesions in the endometrial microenvironment (117).

*Micrococcus*, a Gram-positive bacterial genus classified under the phylum Actinomycetota, naturally colonizes the human skin as part of the commensal microbiota. In recent years, research has uncovered potential associations between an increased abundance of *Micrococcus* and various types of cancer, including cervical cancer (122) and colorectal cancer (123). A recent groundbreaking study has found a positive correlation between the abundance of *Micrococcus* in the endometrial microbiome and the mRNA expression levels of pro-inflammatory cytokines IL-6 and IL-17. This discovery provides compelling evidence supporting a close relationship between *Micrococcus*, the immune system, and EC (117).

In addition, the decline in estrogen levels in vaginal epithelial cells, whether resulting from natural menopause, interruption of hormonal therapy, or cytotoxic cancer treatment outcomes, triggered a shift in the vaginal microbiome towards a non-*Lactobacillus*-dominated microbiome. As a result, there was an increase in the abundance of anaerobic bacteria such as *Gardnerella* and *Atopobium*. An overgrowth of facultative or obligate anaerobic bacteria in the FRT can result in microbial dysbiosis, thereby adversely impacting the healthy vaginal environment (122). Macrophage inflammatory protein (MIP-3α), also known as the chemokine CCL20, has the ability to infiltrate various types of immune cells in the tumor microenvironment (124), including dendritic cells (DCs), regulatory T lymphocytes (Tregs), and Th17 helper cells (125). In studies on EC, scholars have observed an upregulation in the expression of CCL20 and have highlighted its ability to accelerate invasion and induce epithelial-mesenchymal transition (EMT) in EC cells (126, 127). Furthermore, the presence of *Atopobium* in the vagina was associated with the activation of various pro-inflammatory cytokines, including tumor necrosis factor alpha (TNF-α), MIP-3α, and the transcription factor NF-KB (123, 128). Short-chain fatty acids (SCFAs) within the vagina contributed to the development of a pro-inflammatory environment. In cases of vaginal dysbiosis, elevated concentrations of SCFAs increases can enhance the production of IL-8 and TNF-α induced by TLR2 and TLR7 ligands. SCFAs can also facilitate the generation of pro-inflammatory cytokines through partially inducing the production of reactive oxygen species (ROS) (129, 130), thereby exacerbating the inflammatory process and potentially promoting tumor cell proliferation and metastasis, consequently increasing the risk of EC.

### 7.2 Immune response of the endometrial microbiome in endometrial cancer

**Figure 2:**
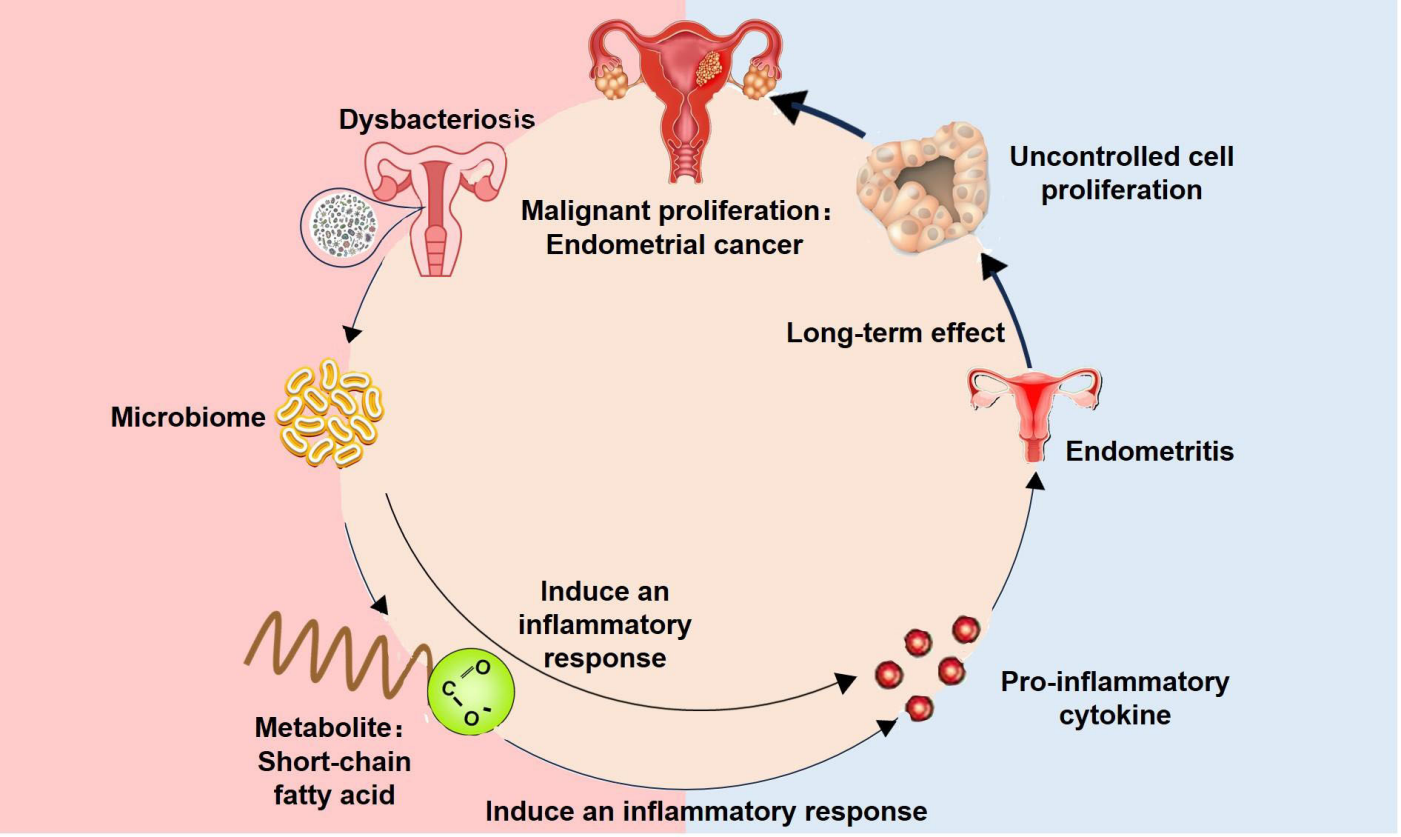
Immune mechanism of endometrial microbiome in the development of endometrial cancer. Dysregulation of the microbial community in the host’s endometrium results in an increase in pathogenic bacteria, which may subsequently induce the release of pro-inflammatory cytokines or toxins. Metabolic products from these pathogens, such as short-chain fatty acids (SCFAs), have the potential to activate the immune system, thereby inducing the release of pro-inflammatory cytokines like TNF-α, IL-1β, IL-6, IL-17, and IL-1α. These pro-inflammatory factors trigger local or systemic inflammatory responses. Prolonged exposure to a pro-inflammatory state within the host’s internal environment can lead to cellular damage. Chronic inflammation and cell proliferation may contribute to abnormal changes and carcinogenesis in endometrial cells. An uncontrolled proliferation of cells ultimately results in abnormal hyperplasia, which may progress into endometrial cancer. Therefore, maintaining microbial homeostasis and preventing the invasion of pathogens are crucial for preserving host health and impeding the onset and progression of cancer. Investigating the relationship between the microbiome and endometrial cancer holds paramount significance in elucidating the pathogenesis of gynecological cancers, particularly endometrial cancer, as well as in developing novel preventive and therapeutic strategies.

In recent years, the investigation of the endometrial microbiome and its intricate interplay with the immune system has garnered significant scholarly attention. The endometrial microbiome maintains a dynamic equilibrium, actively participating in the modulation of immune defense mechanisms in the endometrium. Additionally, their metabolic byproducts exerted influence over crucial physiological processes including endometrial cell proliferation, differentiation, and apoptosis. The maintenance of these functions is crucial to the health of the endometrium. Gynecological cancers originate in the female reproductive organs, and dysbiosis of the microbiota within these organs, particularly imbalances in the endometrial microbiota, can impact the host immune system, thereby increasing the risk of developing EC (**Figure 2**).

## 8 Roles of diverse cytokines in the pathogenesis of endometrial cancer

### 8.1 Interleukin-6 and endometrial cancer

**Figure 3:**
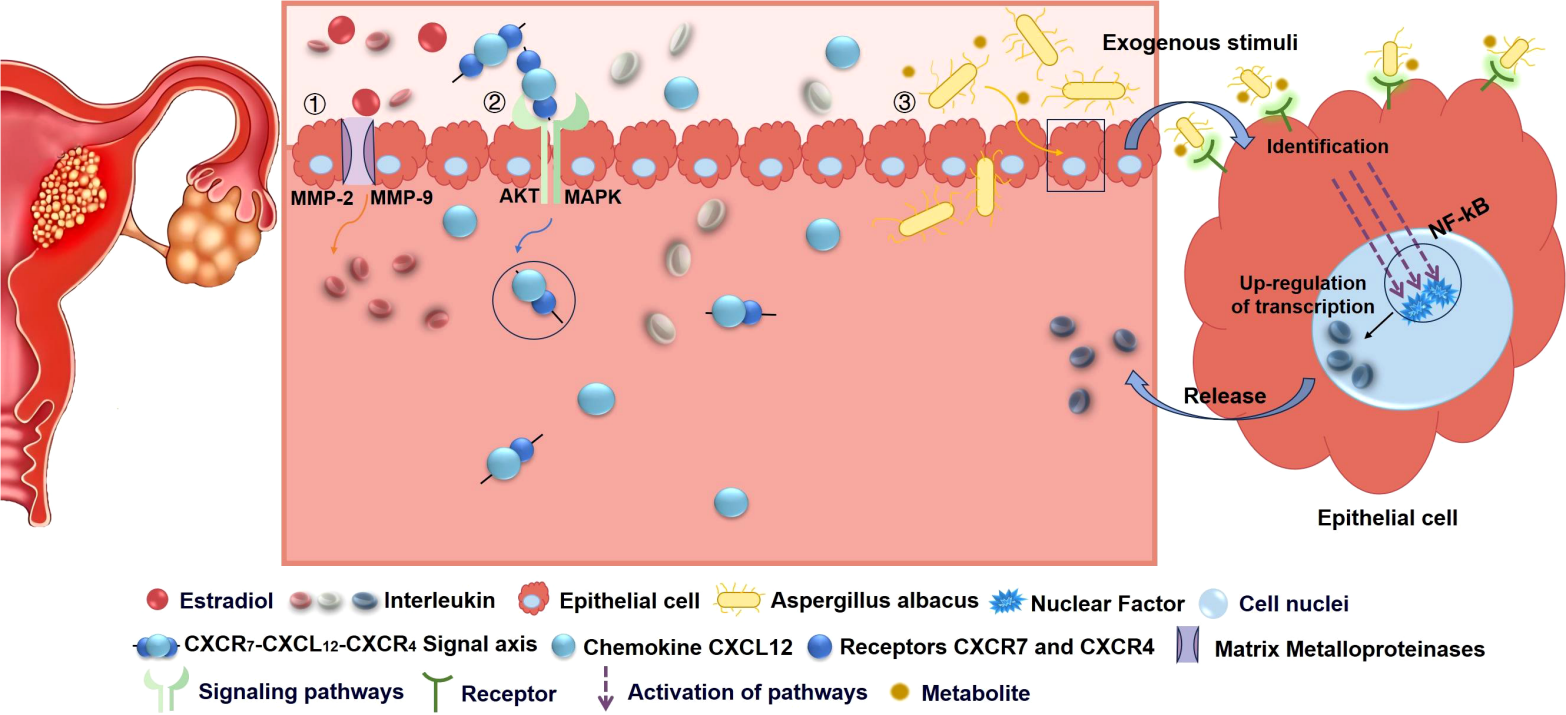
The role of different cytokines in the pathophysiology of endometrial cancer. Diverse cytokines can not only influence cell proliferation and differentiation but also modulate the immune system and inflammatory response, thereby impacting the development of endometrial cancer (1). Interleukin-1β, interleukin-6 and interleukin-8 can trigger inflammation, promote the progression of chronic inflammation, activate signaling pathways, stimulate cell proliferation and immune cell recruitment, and promote the secretion of matrix metalloproteinases to accelerate cancer cell invasion and metastasis (2).The binding of Chemokines to receptor proteins can form signaling axes, which can activate signaling pathways, enhance tumor cell migration, invasion, and angiogenesis, regulate the tumor microenvironment, and participate in the onset of endometrial cancer (3). Bacteria and their metabolites can act as exogenous stimuli, recognized and bound by receptors on cells, transmitting signals to the nucleus to promote the transcription of specific cytokines, thereby influencing the occurrence and development of endometrial cancer.

IL-6, the pivotal member of the IL-6 cytokine family, acts as a soluble factor that stimulates T cells to secrete and modulate B cell responses (131). The signal transduction of IL-6 relies on a hexameric high-affinity complex composed of IL-6, IL-6 receptor α (IL-6Rα), and glycoprotein 130 (gp130), which serves as the shared signaling chain among IL-6 subfamily receptors, thereby facilitating the proliferation of EC cells (132). The production and expression of IL-6 were mainly influenced by ERK-NF-κB signaling pathway (133–135). Matrix metalloproteinases (MMPs) were intimately linked to cancer progression and promote cancer cell invasion and migration by degrading extracellular matrix. Studies demonstrated that IL-6 was involved in estrogen E2-triggered EC cell migration and invasion, accompanied by upregulated expression of MMP-2. Moreover, apart from MMP-2, IL-6 can exacerbate the invasive capacity of EC cells by inducing the release of MMP-9 (136, 137)(**Figure 3**).

### 8.2 Interleukin-8 and endometrial cancer

IL-8 is a major mediator of inflammation and acts as a chemotactic agent for various immune cells populations (138). Studies have shown that an upregulation in the expression level of IL-8 mRNA in EC patients, suggesting a significant role of this cytokine in promoting neutrophils infiltration into tumor tissue (117, 139). In addition, IL-8 exerts its biological effects mainly by binding to its receptors (such as CXCR1 and CXCR2). The expression level of IL-8 receptors may potentially be higher in EC than in normal endometrial tissues. This high expression may enhance the sensitivity of endometrial cancer cells to IL-8, thus promoting the proliferation, invasion and metastasis of tumor cells (138). Studies *in vitro* have found that a*spergillus albacus* can induce NF-κB signaling activity and increase the secretion of IL-8 (140), which may promote the occurrence and development of EC (**Figure 3**).

### 8.3 Chemokines and endometrial cancer

Chemokines are a class of secretory proteins produced by cancer cells and leukocytes infiltrating the tumor microenvironment. These chemokines can regulate cellular behavior by interacting with specific G protein-coupled seven-helix chemokine receptors on the cell surface (141). Notably, chemokines not only participate in regulating the aging process and inducing apoptosis of cancer cells, but also play a crucial role in the progression and metastatic cascade of EC. Specifically, they promote epithelial-mesenchymal transition (EMT), which is a biological process that provides key molecular mechanisms supporting cancer cell invasion and metastasis (142). Numerous studies have robustly demonstrated the pivotal roles of various chemokines in the progression of diverse cancer types, including ovarian, breast, and lung cancers (143–146). Consequently, the expression patterns of chemokines in patients with EC have emerged as a focal point of intense research. In particular, the CXCL12 ligand and its receptors, CXCR4 and CXCR7 form an axis contributing to tumor progression and metastatic cascade of EC (147)(**Figure 3**). CXCL12 exhibited a significant correlation with an unfavorable prognosis across various cancer types and played a pivotal role by activating distinct cellular signaling pathways (141, 147–149). In the context of EC research, the expression level of CXCR4 was significantly upregulated compared to atypical hyperplasia, simple hyperplasia, and normal endometrial cells (150). The data indicated that CXCR4 was detected in up to 69.23% of EC tissue samples (150). Moreover, the expression status of CXCR7 was closely associated with reduced overall survival rates across diverse cancer patient populations (151, 152). Collectively, the intricate CXCL12-CXCR4-CXCR7 signaling axis is emerging as a pivotal predictor for early diagnosis and assessment of unfavorable prognosis in EC. A profound understanding of the molecular mechanisms underpinning this axis is of paramount importance.

However, the precise expression profiles of these chemokines throughout different stages of EC remain undetermined (153). The CCL2-CCR2 signaling pathway plays a pivotal role in the intricate processes of cancer invasion within the tumor microenvironment and lymphatic dissemination (154, 155). A key function of CCL2 is its ability to elicit an invasive phenotype in cancer cells and recruit monocytes to the tumor site, thereby implying that overexpression of CCL2 may have therapeutic implications in gynecological malignancies (156, 157). For instance, the expression of CCL2 served as a biomarker in cervical cancer, while an upregulation of CCR2 expression had been associated with reduced overall survival rates (158). The expression of CCL2 in breast cancer had been identified as an independent risk factor for disease-free survival (DFS) and was significantly correlated with a poor prognosis (159).

### 8.4 Other cytokines and endometrial cancer

The expression of IL-17A within EC tissues has been recently discovered to exhibit a significant elevation, suggesting its potential as a crucial pro-inflammatory factor that promotes endometrial carcinogenesis (160). IL-17A has been confirmed as a vital CD4 T cell-derived pro-inflammatory cytokine, which significantly contributes to tumor angiogenesis, cell proliferation, and invasion in various solid tumors, including breast and cervical cancers (161–163). Tumor necrosis factor alpha (TNF-α), a potent inflammatory cytokine, not only augments local estrogen synthesis in endometrial cells but also stimulates the proliferation of human endometrial cells through adipocyte paracrine mechanisms (164). This aforementioned observation indirectly underscores a potential causal link between obesity and the development of EC. IL-1ra may play a pro-inflammatory role, thereby implying its potential involvement in the promotion of endometrial cancer development (165–167). However, an experimental study has provided empirical evidence supporting the rationality of IL-1ra’s mechanism in playing a protective role against cancer. The results demonstrated that interleukin-1 receptor antagonist (IL-1ra/IL1RN) was negatively correlated with both the overall risk of EC (r=0.86, 95% CI:0.80-0.93, *P*=2.23×10^−4^) and the risk of endometrioid subtype cancer (r=0.85, 95% CI:0.78-0.94, *P*=7.9×10^−4^). And a positive correlation was observed between interferon-induced monokine (MIG/CXCL9) and the risk of non-endometrioid endometrial cancer (r=3.73, 95% CI:1.86-7.47, *P*=2×10^−4^) (168). The presented compelling evidences establishes a correlation between cytokines and EC, but further verification and in-depth investigation into the underlying mechanisms are still needed.

## 9 Conclusions

EC is one of the most common gynecological malignancies, and its incidence is increasing year by year. Obesity, diabetes, PCOS, and other related conditions are all significant risk factors for EC. The reproductive tract microbiome plays a crucial role in maintaining local immune homeostasis. Once the balance of the microbiome is destroyed, it may trigger abnormal immune responses and promote the occurrence and development of EC. Cytokines, being pivotal immunomodulatory molecules, not only regulate tumor cell proliferation, apoptosis and migration but also affect immune cells function and angiogenesis within the tumor microenvironment. Due to the biological heterogeneity of EC, reaching a unified consensus on its molecular subtypes remains a great challenge. Given the intricate interactions between the microbiome and EC, the mechanisms by which specific microbiome and its metabolites regulate the immune microenvironment and thus affect tumorigenesis remain to be further elucidated. Combining microbiome data with multi-omics information, including genomics, proteomics, and transcriptomics data, may provide a more precise way to predict the risk of EC onset and recurrence. In the future, it is worthwhile to explore targeted interventions targeting specific microbiota and evaluate their impact on the therapeutic efficacy of EC. EC research is full of opportunities and challenges. We eagerly look forward to further breakthroughs in this area in the coming years.
